# Editorial: Intermittent Fasting – Mechanisms and Clinical Usefulness

**DOI:** 10.3389/fendo.2021.757539

**Published:** 2021-09-14

**Authors:** Andreas Fritsche, Julia Szendrödi, Annette Schürmann

**Affiliations:** ^1^Medical Clinic IV, University of Tübingen, Tübingen, Germany; ^2^Institute for Diabetes Research and Metabolic Diseases of the Helmholtz Center Munich at the University of Tübingen, Tübingen, Germany; ^3^German Center for Diabetes Research (DZD), Neuherberg, Germany; ^4^Department of Internal Medicine 1 and Clinical Chemistry, Heidelberg University Hospital, Heidelberg, Germany; ^5^Department of Experimental Diabetology, German Institute of Human Nutrition Potsdam-Rehbruecke, Nuthetal, Germany

**Keywords:** intermittent fasting, metabolic syndrome, Parkinsons disease, type 1 diabetes, type 2 diabetes

For more than a decade, intermittent fasting has been carried out by an increasing number of people not only to reduce body weight and improve metabolism, but also as an adjunctive treatment to enhance therapeutic responses. The different forms of intermittent fasting (periodic fasting, time-restricted eating, alternate day fasting and fasting mimicking diet) induce an improvement of energy utilization and mitochondrial function, optimize stress responses, promote repair mechanisms, induce autophagy, etc. (1).

Six publications (two clinical trials, one mini review, two systemic reviews, and one opinion) on the specific Research Topic ‘Intermittent fasting - mechanisms and clinical usefulness’ are published in Frontiers of Endocrinology.

In a small, non-randomized clinical trial performed in Pakistan, the effect of intermittent fasting on lipid metabolism was tested over 6 weeks. The intervention group including 15 mostly overweight individuals fasted 3 days a week for 12 h during day time, whereas the control group did not change the diet. This type of intermittent fasting shows similarity to Ramadan fasting in respect to duration of the fasting period from sunrise to sunset (~12h). However, the Ramadan fasting is done for 4 weeks every day, resulting in 28 intermittent fasting days, whereas in the present study 18 days of intermittent fasting was performed. This amount of intermittent fasting resulted in a body weight reduction of 3 kg, a decrease of LDL cholesterol of 5 mg/dl and an increase of HDL cholesterol of 3 mg/dl. These changes were significantly different to the non-randomized control group suggesting beneficial cardiometabolic effects (Ahmed et al.). However, from a clinical point of view, the effect is rather marginal and needs confirmation in larger long-lasting trials.

For patients with type 1 diabetes (T1D), intermittent fasting may be a therapeutic option, because some of these patients are overweight and display features of the metabolic syndrome. However, fasting is demanding in T1D concerning the control of glucose metabolism. Sometimes fasting may be even very dangerous for individuals with T1D, as this might result in hypoglycaemia or ketoacidosis, depending on the accuracy and success of insulin dose adjustment. Therefore, it is important to test the safety of prolonged fasting periods in T1D. The clinical study examined 20 individuals with T1D who performed two fasting periods. The first period lasted 12 h overnight, the second, prolonged period lasted 36 h starting at 8 PM. The prolonged fasting could be performed safely and did not result in meaningful hypo- or hyperglycemia (Moser et al.). Therefore, future trials are justified to test beneficial effects of intermittent fasting in patients with T1D.

Caloric restriction and (intermittent) fasting have been reported to elicit effects on the physiologic response to ionising radiation. This could be of clinical relevance in the context of patients with cancer undergoing radiotherapy or repetitive medical imaging. Moreover, potential applications could address potential biological countermeasures for the negative consequences of the exposure to ionizing Galactic Cosmic Rays of astronauts conducting deep space exploration missions. The meta-analysis of the current evidence in humans and preclinical models is challenged since the available evidence from controlled clinical trials is small and interventions mostly in addition to radiotherapy also involved immunotherapies, chemotherapy and more. Also such patient populations might be vulnerable to any caloric restriction strategies and various heterogeneous biological responses measured after radiation might serve as outcome measures, not merely metabolic and anthropometric parameters. Nevertheless, the authors here present a balanced review synthesising the evidence from studies with lower evidence and conclusions of their systematic analysis. While included studies did not reveal any benefit from pre-exposure caloric restriction, combination with caloric restriction during and after irradiation might increase the resilience to ionizing radiation (Valayer et al.). Future research is definitely needed in particular upon clinical outcomes in patients undergoing radiotherapy.

Another meta-analysis explored the effects of alternate day fasting, a dietary method characterized by caloric restriction to 25% of daily requirements every other day, followed by one day of *ad libitum* eating. This strategy for weight loss is becoming more popular since calorie counting is only necessary on restricted days, while normal life continues on the other days. Seven RCTs including 269 participants have been included to evaluate the effect of alternate fasting for at least one month on metabolic changes. The authors conclude that alternate day fasting is a safe diet regimen for weight loss with improvement of several cardiovascular risk factors in obese and normal weight humans. However, surprisingly, these improvements are not related to an increase in surrogate parameters of insulin sensitivity or fasting blood glucose, which might limit the clinical application in patients with diabetes (Cui et al.).

Parkinson’s disease (PD) is characterized by the selective loss of dopaminergic neurons of the substantia nigra, inducing motor and neurogenerative symptoms. For the development of Parkinson’s disease, the formation of α-synuclein oligomers, their pathological aggregation in the form of Lewy bodies appear to be harmful. Beside this, mitochondrial dysfunction and oxidative stress play an important role in the PD pathogenesis. Interestingly, lifestyle and genetic factors influencing mitochondrial activity, connectivity and their role in the regulation of apoptosis contribute to the disease and are attractive targets for interventions. In their Opinion article Neth et al. propose an intermittent fasting study with a 16:8 diet for 5 days per week for a period of one year with a 6 months follow-up. The authors provide several arguments pointing towards beneficial effects of this mitochondria-targeting intervention for PD patients: (i) several earlier animal studies e.g. in rodent PD models demonstrated a protection against neuronal excitotoxicity by fasting-induced improvement of mitochondrial function; (ii) anti-diabetic drugs (e.g., Metformin) reduced oxidative stress which was associated with improved motor activity in PD models; (iii) anti-diabetic drugs had beneficial effects in PD patients as it was shown for GLP1 receptor agonist Exenatide (Neth et al.).

Finally, the Mini Review by Schuppelius et al. provides a very broad summary of all clinical trials on time restricted eating (TRE) that were performed in order to reduce body weight, insulin resistance and cardiovascular diseases. Most studies showed positive effects such as lowering blood glucose, improving insulin sensitivity and positivity affecting plasma lipids. Others induced rather no or adverse effects on these traits. The authors tried to find explanations for the controversial findings and conclude that the outcome of TRE approaches depends on an appropriate study design. For this, changes of caloric intake, the fasting duration and the time window of the fasting period *via* affecting the circadian clock appear to have a major impact on the success of this intervention (Schuppelius et al.).

In summary, the publications highlight various aspects of intermittent fasting and raise more follow-up questions than they provide conclusive answers. That is a good sign as this is often the case with current scientific topics.

## Author Contributions

AF, JS and AS wrote the editorial together. All authors contributed to the article and approved the submitted version.

## Funding

German Federal Ministry of Education and Research [BMBF, DZD grants 01GI0925, 82DZD00702, 82DZD00302].

## Conflict of Interest

The authors declare that the research was conducted in the absence of any commercial or financial relationships that could be construed as a potential conflict of interest.

## Publisher’s Note

All claims expressed in this article are solely those of the authors and do not necessarily represent those of their affiliated organizations, or those of the publisher, the editors and the reviewers. Any product that may be evaluated in this article, or claim that may be made by its manufacturer, is not guaranteed or endorsed by the publisher.
